# COVID‐19 vaccination status and testing rates in Finland—A potential cause for bias in observational vaccine effectiveness analysis

**DOI:** 10.1111/irv.12993

**Published:** 2022-04-27

**Authors:** Ilari Kuitunen, Mikko Uimonen, Santeri J. Seppälä, Ville T. Ponkilainen

**Affiliations:** ^1^ Institute of Clinical Medicine University of Eastern Finland Kuopio Finland; ^2^ Mikkeli Central Hospital Mikkeli Finland; ^3^ Central Finland Hospital Nova Jyväskylä Finland

**Keywords:** COVID‐19, epidemiology, surveillance, vaccination

## Abstract

COVID‐19 vaccination effectiveness has been monitored in observational studies (test‐negativity design or traditional cohort design), but these studies have not addressed the potential behavioral bias between vaccinated and unvaccinated individuals. We aimed to address this by comparing COVID‐19 testing rates between vaccination status and whether vaccination changes the testing rates. We found that three times vaccinated had least tests performed during the pandemic and unvaccinated had the highest testing rate. Each vaccination dose increased the testing rate. In conclusion the observational studies addressing vaccine effectiveness should also present testing rates between vaccinated and unvaccinated to address the potential behavioral bias.

## 1 BACKGROUND

COVID‐19 vaccinations had all excellent efficacy against severe disease (i.e., hospitalization, intensive care unit [ICU] admission, or death) in the original randomized controlled trials run by the vaccine developers, and those studies also showed protection against the SARS‐CoV‐2 virus infection.^1^, ^2^, ^3^ Alongside these trials, real life observational data have been used to monitor the vaccine efficacy continuously.^4^, ^5^, ^6^ As the observational studies are prone to certain biases, test‐negativity design has been used to monitor the vaccine efficacy against infection (especially popular previously in influenza vaccinations), in which the vaccine rates between those who tested positive are compared to those who tested negative.^7^, ^8^ Although this design acknowledges some of the biases in the more traditional vaccinated versus unvaccinated cohort/case–control designs, it is still vulnerable to behavioral bias.^7^ In order to work properly, test‐negativity design would need the vaccinated and unvaccinated samples to have similar healthcare seeking behavior. Thus, if either group does not seek for testing in symptoms, it may lead to neglected selection bias in the sample.^7^, ^9^ This bias is not seen when the outcome is objective, such as ICU admission or death, but it may cause bias, if only infection and detection rates are measured.^9^ We aimed to assess the potential bias due to the healthcare seeking behavior related to COVID‐19 vaccination by comparing testing rates between vaccinated and unvaccinated population.

### 1.1 Materials

We conducted an observational retrospective analysis in Southern Savonia region of Finland. Our study period was from August 1, 2020, to January 31, 2022. During this period, all people in this area had the possibility to book COVID‐19 polymerase chain reaction (PCR) testing free of charge either by phone call or via internet. The testing guidelines were practically unchanged during this period as the recommendation was to test everyone with the slightest symptom of COVID. Testing was recommended if any of the following symptoms were found: Fatigue, headache, fever, cold, cough, difficulty in breathing, vomiting, or diarrhea.

We included all citizens aged 18 or more in our region. We generated the study groups according to vaccination status on January 31, 2022. The vaccinations began on January 1, 2021 and were then first given to healthcare personnel and then shortly to those with highest risk of COVID‐19. Mass vaccinations for all citizens aged 18 or more began in April 2021, and all the citizens in our area had had the possibility to have three (four for immunosuppressed) doses before the January 31, 2022.

The data from three sources were combined by using the patient ID: (1) The date and number of received vaccines, (2) the date of the COVID‐PCR‐tests that patient underwent, and (3) the results of the COVID‐PCR‐tests.

We then analyzed the testing rates and test‐positivity rates between the groups and stratified the groups between the vaccination status from zero to four times vaccinated. Vaccination status was defined as the number of vaccines taken before January 31, 2022. The comparisons were conducted according to this status retrospectively.

The incidence of tests per week was calculated as the test rate per week divided by the patients with the same vaccination status.

A linear mixed effect model was used to evaluate the timing of the tests compared to the vaccination status of the patients to investigate if the number of received vaccines affected how often the patients underwent tests. To adjust for the changes in the severity of the pandemic and changing test policies, we included the delay between the start date of the study and the current time‐interval in the model. The data for the model were in a format where each time‐interval between the consecutive vaccinations was used as separate cases. To obtain comparable test rates, the number of underwent tests per time‐interval was handled as tests per week rate. The number of received vaccines and time‐interval was included as fixed factors. Patients, and interaction between delay from the start and the number of vaccines as random factors. The results of the mixed‐model were interpreted as regression coefficient with 95% confidence intervals (CI).

All analyses were performed using R version 4.0.5 (R Foundation for Statistical Computing, Vienna, Austria).

Ethical committee evaluation was waived according to the Finnish research laws as we conducted a retrospective register‐based study and used routinely recorded data. We have the research permission of Southern Savonia Health Officials, granted by the Medical Director, to access and analyze the data.

## 2 RESULTS

**FIGURE 1:**
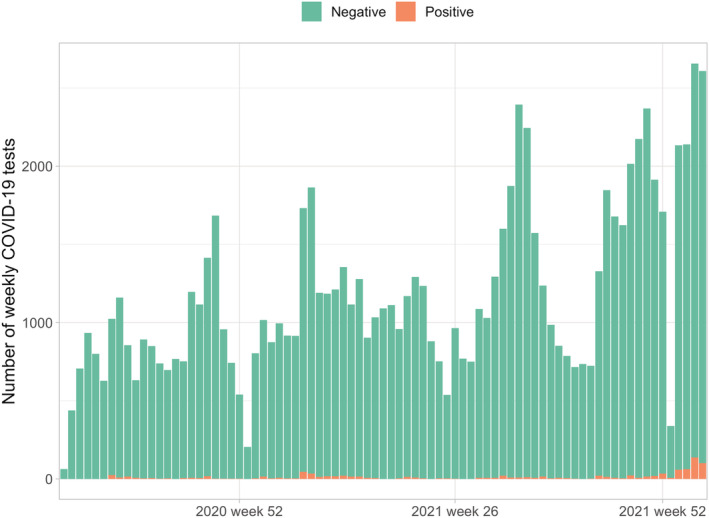
Weekly number of COVID‐19 polymerase chain reaction (PCR) tests and positive findings from August 2020 to January 2022 in Southern Savo region of Finland

**FIGURE 2:**
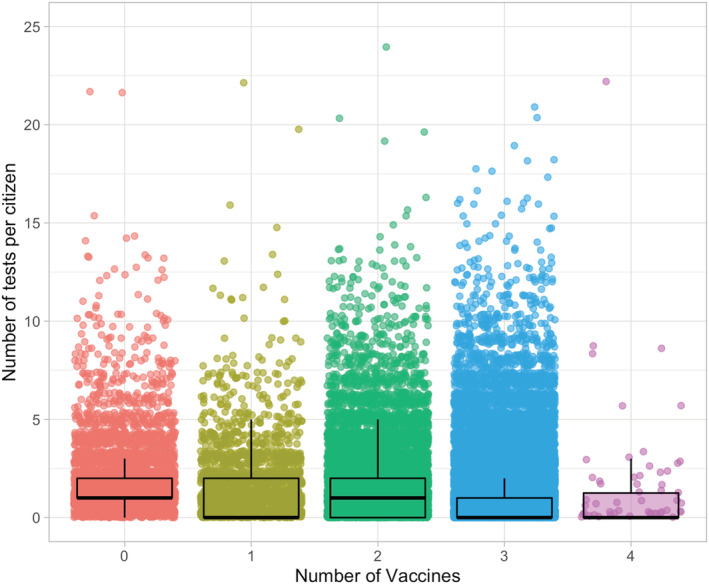
Number of tests per individuals stratified by the vaccination status

A total of 63,339 citizens were included for analysis and of these 4455 were unvaccinated. Most of the included citizens (*n* = 43 090, 68%) were three times vaccinated. We had information of 108,138 tests available, and of these 2401 were positive. The testing increased throughout the study period and hit the record rates in January 2022 and positive findings followed similar trend (Figure 1). The overall testing rate was highest among those unvaccinated and lowest among three times vaccinated (Figure 2). In the unvaccinated group 14.1% were not tested during our study period, the respective rates were 48.3% among one time vaccinated, 41.6% among two times vaccinated, 57.2% among three times vaccinated, and 56.0% among four times vaccinated. A total of 1.4% (*n* = 628) (1% of the population included in the study) had 0 tests and 0 vaccinations.

**FIGURE 3:**
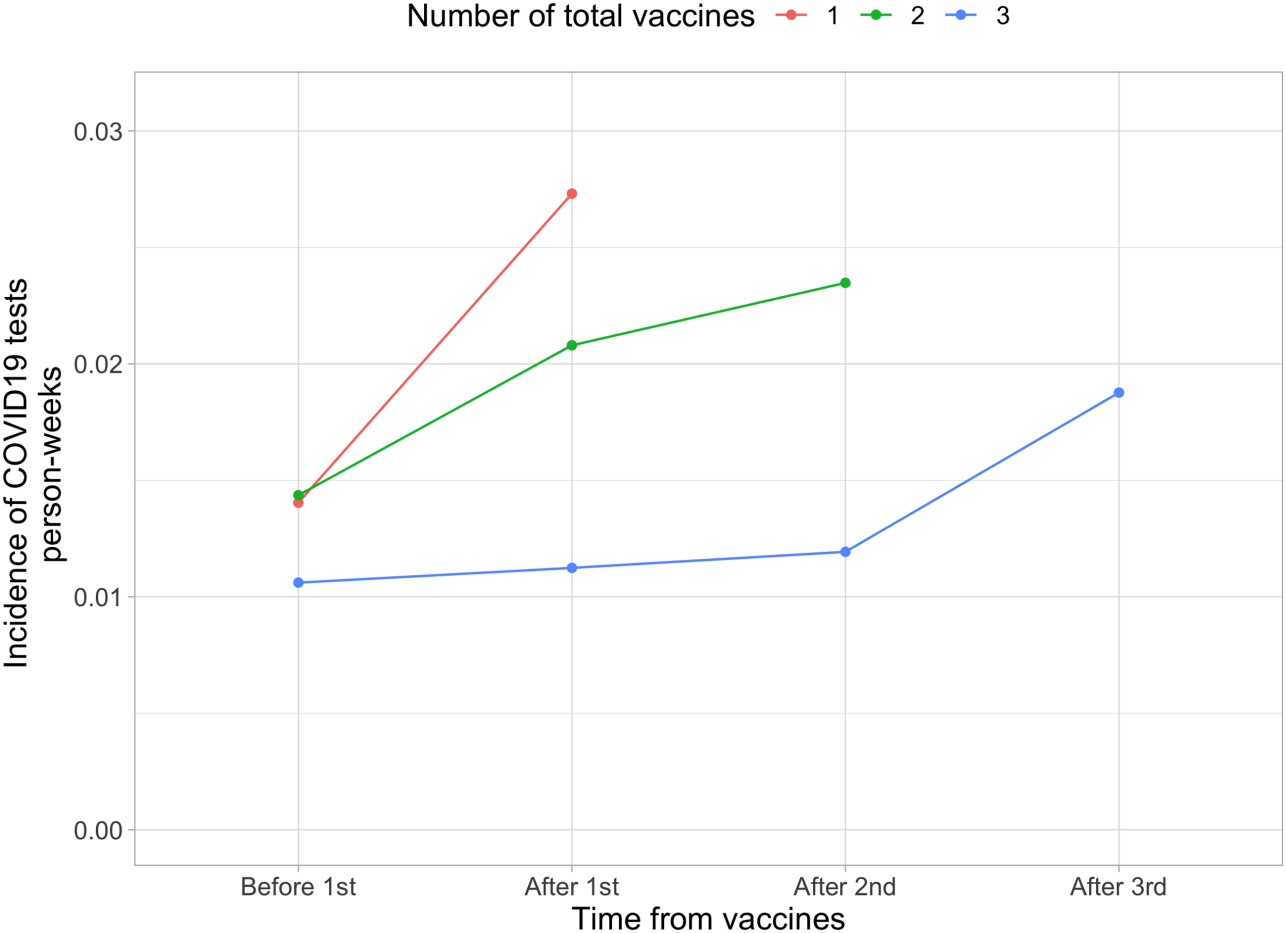
COVID‐19 testing incidence prior and after vaccination doses stratified by the final vaccination status. Fourth dose was left out of this analysis due to the low number of individuals

The linear mixed model showed that every received vaccine increased the test rate per week by 0.0015 (95% CI 0.0080–0.0023). Thus, the testing behavior seemed to change with the vaccination status as the testing increased after each vaccination dose (Figure 3).

## 3 DISCUSSION

We found that over half of the three times vaccinated had not had a single COVID‐19 PCR test performed. This indicates that either the vaccination reduces the symptomatic SARS‐CoV‐2 infections, or that individuals rely on the vaccine so much that they do not see the testing necessary despite the recommendations. However, in the assessment of the testing rates in relation to vaccination, the testing rates increased after received vaccinations in each group. Still, it seems evident that those vaccinated three times had the lowest testing rate, which should be taken into account in the vaccine efficacy analyses.

Initially, the vaccination was hoped to reduce the possible viral spreading, but during the omicron period, it became clear fast that the vaccinated individual may spread the disease and get infected as well as those unvaccinated.^10^ Three doses though protect still from serious events (ICU admission and death).^11^ The largest test‐negativity study from England addressed the vaccination status against symptomatic omicron infection and showed low protection with two doses and better protection after booster shots, but it did not analyze the healthcare seeking behavior.^12^ Most likely this will lead to bias in the results and overestimate the booster shot effectiveness, as those having booster could have the lowest testing rate based on our results and therefore the vaccine effectiveness results might be due to reduced testing in this group instead of true effect.

We have some limitations to our data. First, we do not have the numbers of home testes performed. These have been recommended in our area since September 2021, but all of the positive home test findings are guided to be confirmed in an official PCR test. A second limitation is the lack of testing data on private sector but based on the reports published in local newspaper the private testing capacity in our region has been less than 5% of the publicly funded. Third limitation is that we do not know the symptoms which lead to the test. It may be that those vaccinated have got tested in order to get their COVID‐19 passport eligible for example to traveling (although this was not officially allowed indication for free testing).

The main strengths are that the testing has been free for citizens and the testing time can have been booked directly from internet. Testing guidelines have remained practically unchanged in our region during our study period. Majority of the regions in Finland stopped the testing of vaccinated individuals already in September 2021 and stopped the testing of mild cases in December 2021 or January 2022. Furthermore, our region has the highest vaccination coverage in the country, and we have had the lowest COVID‐19 incidence from August 2020 to the mid‐January 2022, although our region has had one of the highest testing rates during this time period.

## 4 CONCLUSION

When conducting a test‐negativity designed study to analyze vaccine efficacy against infection based on observational data, the testing rates in the selected population should be presented, as there might be substantial behavioral differences in the groups, which may cause selection bias for the study cohorts and therefore bias the efficacy estimated. In our example the results in a classic test‐negativity design would have overestimated the vaccine efficacy against COVID‐19 infection due to the substantially lower testing rates among vaccinated individuals. The test‐negativity design is most suitable when the outcome does not depend on the patient's behavior (e.g., ICU admission or death).

## AUTHOR CONTRIBUTIONS

**Ilari Kuitunen:** Conceptualization; data curation; investigation; validation. **Mikko Uimonen:** Conceptualization; funding acquisition; methodology; project administration; software; supervision. **Santeri Seppälä:** Conceptualization; funding acquisition; project administration; resources; supervision. **Ville Ponkilainen:** Conceptualization; data curation; formal analysis; investigation; methodology; validation; visualization.

5

### PEER REVIEW

The peer review history for this article is available at https://publons.com/publon/10.1111/irv.12993.

## References

[1] Polack FP , Thomas SJ , Kitchin N , et al. Safety and efficacy of the BNT162b2 mRNA Covid‐19 vaccine. N Engl J Med. 2020;383(27):2603‐2615. doi:10.1056/NEJMoa2034577 33301246 PMC7745181

[2] Baden LR , El Sahly HM , Essink B , et al. Efficacy and safety of the mRNA‐1273 SARS‐CoV‐2 vaccine. N Engl J Med. 2021;384(5):403‐416. doi:10.1056/NEJMoa2035389 33378609 PMC7787219

[3] Sadoff J , Gray G , Vandebosch A , et al. Safety and efficacy of single‐dose Ad26.COV2.S vaccine against Covid‐19. N Engl J Med. 2021;384(23):2187‐2201. doi:10.1056/NEJMoa2101544 33882225 PMC8220996

[4] Haas EJ , Angulo FJ , McLaughlin JM , et al. Impact and effectiveness of mRNA BNT162b2 vaccine against SARS‐CoV‐2 infections and COVID‐19 cases, hospitalisations, and deaths following a nationwide vaccination campaign in Israel: an observational study using national surveillance data. Lancet. 2021;397(10287):1819‐1829. doi:10.1016/S0140-6736(21)00947-8 33964222 PMC8099315

[5] Bar‐On YM , Goldberg Y , Mandel M , et al. Protection of BNT162b2 vaccine booster against Covid‐19 in Israel. N Engl J Med. 2021;385(15):1393‐1400. doi:10.1056/NEJMoa2114255 34525275 PMC8461568

[6] Dagan N , Barda N , Kepten E , et al. BNT162b2 mRNA Covid‐19 vaccine in a nationwide mass vaccination setting. N Engl J Med. 2021;384(15):1412‐1423. doi:10.1056/NEJMoa2101765 33626250 PMC7944975

[7] Jackson ML , Nelson JC . The test‐negative design for estimating influenza vaccine effectiveness. Vaccine. 2013;31(17):2165‐2168. doi:10.1016/j.vaccine.2013.02.053 23499601

[8] Chua H , Feng S , Lewnard JA , et al. The use of test‐negative controls to monitor vaccine effectiveness: A systematic review of methodology. Epidemiology. 2020;31(1):43‐64. doi:10.1097/EDE.0000000000001116 31609860 PMC6888869

[9] Lewnard JA , Patel MM , Jewell NP , et al. Theoretical framework for retrospective studies of the effectiveness of SARS‐CoV‐2 vaccines. Epidemiology. 2021;32(4):508‐517. doi:10.1097/EDE.0000000000001366 34001753 PMC8168935

[10] Brandal LT , MacDonald E , Veneti L , et al. Outbreak caused by the SARS‐CoV‐2 omicron variant in Norway, November to December 2021. Euro Surveill. 2021;26(50):2101147 doi:10.2807/1560-7917.ES.2021.26.50.2101147 PMC8728491 34915975

[11] Kuhlmann C , Mayer CK , Claassen M , et al. Breakthrough infections with SARS‐CoV‐2 omicron despite mRNA vaccine booster dose. Lancet. 2022;399(10325):625‐626. doi:10.1016/S0140-6736(22)00090-3 Epub 2022 Jan 18 35063123 PMC8765759

[12] Andrews N , Stowe J , Kirsebom F , et al. Covid‐19 vaccine effectiveness against the omicron (B.1.1.529) variant. N Engl J Med. 2022;NEJMoa2119451 doi:10.1056/NEJMoa2119451 Epub ahead of print PMC8908811 35249272

